# Reconsidering the Specialist-Generalist Paradigm in Niche Breadth Dynamics: Resource Gradient Selection by Canada Lynx and Bobcat

**DOI:** 10.1371/journal.pone.0051488

**Published:** 2012-12-07

**Authors:** Michael J. L. Peers, Daniel H. Thornton, Dennis L. Murray

**Affiliations:** 1 Department of Biology, Trent University, Peterborough, Ontario, Canada; 2 Panthera, New York, New York, United States of America; Institut Mediterrani d'Estudis Avançats (CSIC/UIB), Spain

## Abstract

The long-standing view in ecology is that disparity in overall resource selection is the basis for identifying niche breadth patterns, with species having narrow selection being classified “specialists” and those with broader selection being “generalists”. The standard model of niche breadth characterizes generalists and specialists as having comparable levels of overall total resource exploitation, with specialists exploiting resources at a higher level of performance over a narrower range of conditions. This view has gone largely unchallenged. An alternate model predicts total resource use being lower for the specialized species with both peaking at a comparable level of performance over a particular resource gradient. To reconcile the niche breadth paradigm we contrasted both models by developing range-wide species distribution models for Canada lynx, *Lynx canadensis*, and bobcat, *Lynx rufus*. Using a suite of environmental factors to define each species’ niche, we determined that Canada lynx demonstrated higher total performance over a restricted set of variables, specifically those related to snow and altitude, while bobcat had higher total performance across most variables. Unlike predictions generated by the standard model, bobcat level of exploitation was not compromised by the trade-off with peak performance, and Canada lynx were not restricted to exploiting a narrower range of conditions. Instead, the emergent pattern was that specialist species have a higher total resource utilization and peak performance value within a smaller number of resources or environmental axes than generalists. Our results also indicate that relative differences in niche breadth are strongly dependent on the variable under consideration, implying that the appropriate model describing niche breadth dynamics between specialists and generalists may be more complex than either the traditional heuristic or our modified version. Our results demonstrate a need to re-evaluate traditional, but largely untested, assumptions regarding resource utilization in species with broad and narrow niches.

## Introduction

Ecological theory explaining niche breadth dynamics of species is founded on basic principles of resource selection, biotic interactions and evolution. It follows that species can either focus their resource choice to exploit a few resources well, or else broaden their choice to use more resources adequately [1]–[3]. The variance in resource use is the metric by which niche breadth is quantified, and although species’ differentiation according to this metric has been questioned [4], the general consensus is that evolution can give rise to individual species with clear differences in niche breadth dimensions [5]. Species with narrow niches, “specialists”, are presumably favoured during periods of environmental stability or homogeneity, whereas those with broad niches, “generalists”, likely are favoured during environmental instability or heterogeneity [5], [6]. It is noteworthy that the evolution of specialists and generalists also may implicate a variety of more specific considerations such as efficiency of food source use [7], [8] or intensity of biotic interactions (e.g. competition and mate choice; [3]), which can affect population density and environmental carrying capacity of the species. Differentiating species according to their relative niche breadth is a common approach in community ecology, giving rise to an understanding of both how a particular species relates to its environment as well as what role it may play in the ecosystem (e.g., [9]–[11]).

**Figure 1:**
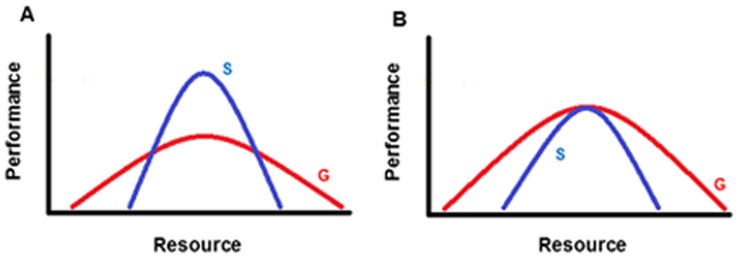
Conceptual model of specialist and generalist responses to resource gradients. A) Standard model; B) Alternative model. The models differ in that the alternative represents environmental restrictions on peak performance and total exploitation by the specialist. The figures are not drawn to scale but rather provide a relative comparison between specialists and generalists.

The difference between specialist and generalist species is commonly illustrated using a simple conceptual model (Fig. 1a, standard model), where specialists have a narrower breadth in resource use than generalists, but within a narrow range of suitable conditions can reach a higher level of performance (e.g., prey capture rate, survival rate, density, etc.). In contrast, generalists have a broader range of used resources, peaking at a lower level of performance [12]–[14]. The qualitative implications of this heuristic are that: 1) the total exploitation of a particular resource (i.e., area under the curve, Fig. 1a) will be approximately the same for each species, but that: 2) maximum exploitation will be higher for the specialist over the narrow range of specialization (i.e., peak height of the curve, Fig. 1a).

This simple but attractive model has gone largely unchallenged, perhaps owing to the substantive difficulties in quantifying and comparing niche breadth between species. Indeed, there is no particular reason why niche breadth restriction through specialization must be accompanied by similar total exploitation as generalist species, or that higher peak exploitation over a narrow range of the resource be characteristic of specialists. For example, Barkae et al. [15] showed that species with a narrow niche breadth do not necessarily achieve a higher level of performance than those with a broader niche within the range of suitable conditions. Others also have failed to find a negative relationship between the breadth of exploited resources and various measures of performance [16], [17]. In fact, species with a narrow niche breadth may perform comparably to others (i.e., have similar peak performance) if, despite specialization, the environment will only support a lower level of exploitation of their preferred resource, or species with a broad niche breadth do not demonstrate an assumed performance trade-off (Fig. 1b, alternative model). Under these circumstances the total performance for the specialist also would be lower, but it could nonetheless persist by maintaining a lower population density. Although the specialist can reach the same level of performance, it can only be accomplished for a smaller breadth of the resource and therefore exits at lower densities. Accordingly, the standard model for niche breadth may not accurately reflect niche characteristics of many species and it logically follows that there is a need to refine our understanding of the relative shape and dimensions of resource use and associated performance by species, to better understand such fundamental underpinnings in community ecology.

The majority of studies examining species niche breadth have compared responses along one or two resource or environmental gradients (e.g. habitat type, prey composition; [15], [18]). However, a more sophisticated understanding of the specialist/generalist distinction should require evaluation along multiple dimensions of environmental and resource conditions. Further, it is critical that to accurately quantify niche breadth, the full spectrum of resource use must be considered. However, too often such efforts have included niche analysis considering only a portion of a species’ range, which precludes the ability to fully document the spectrum of utilization. Species distribution models (SDMs) offer the opportunity for quantifying species’ niche breadth by relating records of species occurrence to a suite of environmental variables and developing projections of suitable habitat [19]. SDMs can serve to examine broad-range relationships between environmental suitability and utilization [20], and recently have been widely used to categorize differences in niche breadth of species [21], [22]. Although SDMs often focus on understanding responses of species to coarse-scale climatic conditions that may only indirectly relate to resource gradients, such models have been used to successfully predict distribution patterns of species at both low and high trophic levels [23]–[25]. This suggests that these environmental gradients are reflective of resource gradients across many species [26], although we recognize that this is an assumption that may not always be met [27]. This also highlights the close association even between species at a higher trophic level and the environmental conditions that they occupy, and emphasizes that SDMs can serve to infer niche dimensionality under the assumption that habitat suitability or probability of occurrence is correlated with species performance [28], [29].

In this study, we investigate the range-wide niche breadth and environmental responses of two closely related species, Canada lynx (*Lynx canadensis*) and bobcat (*Lynx rufus*). Previous research at smaller scales suggests that these two species may differ in their ability to use dietary and habitat resources; Canada lynx are specialist predators of snowshoe hare [30], while bobcats prey on a wide variety of food sources from hares, rodents, and even ungulates (e.g., [31]–[34]). Canada lynx tend to select boreal and montane coniferous forested areas that are more suitable to their primary food source [35], [36], whereas bobcats exploit a range of ecotypes including subtropical swamps, arid landscapes and temperate forests [37]. Morphologically, Canada lynx are advantaged for hunting snowshoe hares given their low foot-load and long legs allowing them to move in deep and soft snow [38]; such habitats are less suitable to bobcats. Accordingly, differences in niche breadth between these two species should be apparent, but the fact that both occur extensively across North America leaves open the question of whether their niche dimensionality conforms to the standard specialist-generalist model. Through an assessment of niche breadth metrics and responses to environmental variables, we use lynx and bobcat to test between the two hypotheses of niche dimensionality (Fig. 1). We predicted that, given the multiple natural constraints (ex. snow, altitude, and temperature) acting on performance and the lower population density of lynx relative to bobcats, our alternate model (Fig. 1b) of niche breadth dynamics would conform more closely to patterns expressed by these two felid species.

## Materials and Methods

### Species Data

We obtained data on Canada lynx and bobcat presence across North America using museum and harvest records. Museum records from freely accessible databases (MaNIS; www.manisnet.org, and CONABIO; www.conabio.mx) were utilized, as well as data from several smaller museums that provided data through individual contact (see Table S1 for list). Specimen records from museums provided a locality description of the specimen along with the date of collection. We converted locality descriptions to X/Y coordinates with an associated uncertainty using the program Biogeomancer [39]. During this step, localities with large uncertainty levels (>13 km radius of uncertainty) were eliminated. Localities collected before 1940 were removed from the data set, to improve the accuracy of the distribution models [40] and to better match the time frame of our environmental data (see below). Presence records for the two species in Canada were also obtained from provincial harvest records (see Table S1 for list). Harvest records had varying levels of uncertainty, with some localities providing fine resolution data for trapping location (i.e., registered trapline), while others offered more coarse resolution (i.e., township or county). As with the museum records, we excluded from further consideration all records with >13 km uncertainty. To accommodate recent range shifts in Canada lynx, we also included a subset of data on historic lynx occurrence in the United States based on sightings, museum records, and other sources (see [41]). For each carnivore species, three separate datasets of presence records were created across the range of both species, representing different levels of uncertainty. These uncertainty levels corresponded to grid cells of 10****km^2^, 15****km^2^ and 20****km^2^. Species ranges were determined using Natureserve (http://services.natureserve.org); Natureserve is an open access organization that provides information on the distributions and abundance of species. Ranges were adjusted for presence records that fell outside of the range provided from Natureserve. Results of subsequent analyses from all three uncertainty levels were qualitatively similar, and here we present only the results of analyses based on presence records at a mid-level of uncertainty (15****km^2^) because these produced the most reliable distribution models. However, models generated using 10****km^2^ and 20****km^2^ grid cells yielded qualitatively similar results (see Fig. S3, FS4, FS5, Fig. S6).

### Environmental Data

Range-wide SDM comparison for mesocarnivores occupying most of North America requires the use of niche metrics that are common to both species. Because lynx and bobcat both use a variety of prey and habitat types and do not overlap fully across their range, we related SDMs to select climatic variables that should correspond to prey or habitat requirements for each species. Climatic variables were obtained from the WorldClim database [42], which provides a variety of climatic data averaged over the years 1950–2000; we used the 19 bioclimatic variables in modeling. An altitude layer was also acquired from the WorldClim database. In addition, we calculated long-term (1979–2000) average winter (October-March) snow depth and snow cover using data from the North America Regional Reanalysis dataset [43], because we expected *a priori* that snow variables would have a strong influence on distributions of these two mesocarnivores. We also included information regarding the ecoregion of each grid cell (United States Environmental Protection Agency, see [44]). All environmental data were resampled to correspond to the three grid cell sizes used to account for varying uncertainty in the presence records (10, 15, and 20****km^2^). Given the large number of potential environmental variables (particularly, the bioclimatic variables), we performed an initial screening by running MaxEnt models [20] for both species using all available variables. We then eliminated variables that had a small relative influence (i.e. not one of the top 10 most influential variables for either species). From this reduced set of environmental variables, we calculated the correlation coefficients of all variables and eliminated variables that were strongly correlated (r >0.85) with other variables. When faced with a pair of highly correlated variables, we chose to retain the variable that was most biologically meaningful. In total, six bioclimatic variables were used in the final MaxEnt modeling (maximum temperature of the warmest month, minimum temperature of the coldest month, temperature seasonality, precipitation of the warmest quarter, precipitation of the coldest quarter and mean diurnal range), as well as snow depth, snow cover, ecoregion, and altitude.

### Model Development

The program MaxEnt was used to create SDMs for each species. MaxEnt compares presence records with randomly selected points from the background to create maps of habitat suitability and determine the effect of environmental variables on species presence. MaxEnt assumes that sampling of presence locations is unbiased, and biased sampling promotes model inaccuracy [20]. Presence records obtained from museum samples can be biased given collection patterns favouring areas near roads and higher human density [45]. Use of harvest records adds additional uncertainty owing to jurisdictional differences in location uncertainty. We initially sought to reduce the unevenness in density of presence records by subsampling our presence records so that only one record was included for every 900****km^2^ area. Bias in presence records was further addressed by creating a bias grid for use in MaxEnt modeling, following procedures outlines in Elith et al. [46]. The bias grid is used to down-weight the importance of presence records from areas with more intense sampling (i.e., areas with a high density of presence records; [46]). The weighting surface is calculated based on the number of presence records within a neighborhood around any given cell (weighted by a Gaussian kernel with a standard deviation of 200****km). The weighting surface was then scaled to a maximum of 20 and minimum of one to avoid extreme down-weighting of highly sampled cells (Elith et al. [46]). We developed MaxEnt models for lynx and bobcat using background records selected from the United States, Canada and Mexico. The models were run without the threshold feature (which allows abrupt step-like relationships between response and predictor) to reduce the numbers of estimated parameters and to allow better understanding of the variable response curves for each environmental layer.

Performance of MaxEnt models was calculated based on Receiver Operating Characteristic Plots (ROC) and Area Under the Curve (AUC) statistics. AUC values range from 0 to 1, with the value indicating the probability that a randomly selected presence point will have a higher suitability value from the model than a randomly selected location in the background. We performed a 10-fold cross-validation procedure to create the MaxEnt models and calculate AUC statistics. The average of the 10 models produced during the cross-validation was used to calculate model performance and generate corresponding response curves of niche dimensionality, which indicate how habitat suitability changes as a function of individual environmental predictors in the analysis (see below).

### Testing the Two Relationships

First, we examined if bobcats had greater niche breadth than lynx while considering the full set of environmental variables simultaneously (which constituted an explicit test of whether lynx and bobcats could be considered “generalists” and “specialists”, respectively). We determined niche breadth for each species by using the suitability scores generated from MaxEnt models (which were functions of all environmental variables) to calculate Levin’s concentration metrics (implemented in EMNTools; [47]). Levin’s concentration metric ranges from 0–1 where 0 indicates minimum niche breadth (where only one grid cell in the geographic space has a nonzero suitability) and 1 indicates the maximum (where all grid cells are equally suitable; [22]). Because the two species exist in different geographic areas, differences in the background environment in which they live could drive apparent differences in niche breadth, and it is therefore necessary to account for such disparity in any niche breadth assessment [22], [48]. To do this, we calculated the expected niche breadth of the common background environment for each species by creating 100 replicate datasets of randomly-created presence-points located in the Canada lynx and bobcat ranges. These replicate datasets were used to develop MaxEnt models and to determine the expected niche breadth based on the available environment in each species range. These 100 niche breadth values based on our replicated models formed the null expectation of niche breadth in each carnivore range [22]. The differences between each 100 replicate values of niche breadth and the niche breadth values based on the actual species models were calculated for each species, with positive or negative values indicating that niche breadth was wider or narrower than expected, respectively. We compared these 100 values across species (via a t-test) to determine if the two species differed significantly in the amount of environmental breadth they occupied relative to expected.

We tested the two alternate models of niche dimensionality between the specialist lynx and generalist bobcat (Fig. 1) by examining the probability of occurrence according to each environmental variable. The corresponding response curves reveal the extent of variability in habitat suitability according to each environmental variable. MaxEnt provides two sets of response curves: one showing how habitat suitability changes as a function of a particular environmental variable while holding all other variables at their average value, and another showing how habitat suitability changes as a function of a particular environmental variable when that variable is the only variable under consideration. Due to the presence of meaningful correlations among our response variables, even after pre-screening, we only report the latter response curves but note results were qualitatively similar using both approaches. We integrated across the range of x and y values along each response curve to obtain the total area under each response curve (our index of total resource exploitation). To standardize these area calculations, we divided each area calculation by the range along the x axis for each variable for a range from 0 to 1 (with values closer to 1 indicating a greater total exploitation of that particular variable). This was accomplished for all 10 replicates of the MaxEnt model, and differences in standardized area calculations for each response curve were compared across species. We calculated the estimated peak performance value for each environmental gradient by using the correlated maximum height (i.e., maximum achieved habitat suitability value) given by each replicate, and compared peak performance between species for each variable. We also determined average total exploitation and peak performance for each species by averaging the standardized area calculations and height calculations across all environmental variables. We assume a positive correlation between the MaxEnt habitat suitability and performance of the species (e.g., density), and that this correlation is similar between the two species. MaxEnt models for a variety of species are positively correlated with population density or other performance metrics (e.g., [28]–[29]), and given that presence records of bobcat and lynx were obtained in a similar manner, and the two species have similar trophic levels and life history traits, the correlation between suitability and performance is likely not radically different between the two species. Comparisons of environmental variables between species were conducted via t-test.

## Results

### Species Distribution Models

**Figure 2:**
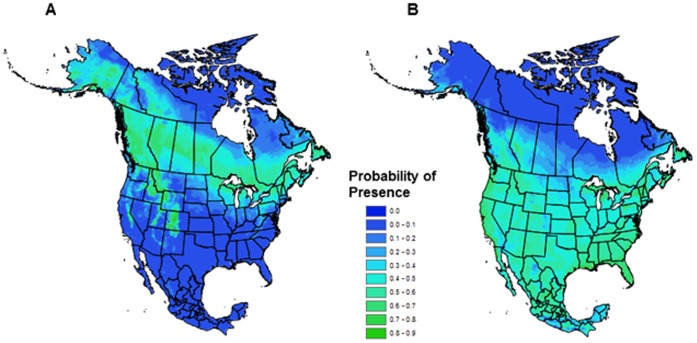
Predicted probability of presence models for lynx and bobcat. Predicted model of occurrence for A) Canada lynx and B) bobcat. Dark green colours represent areas of higher habitat suitability or higher probability of species presence.

After processing lynx and bobcat locations according to the appropriate uncertainty level and excluding points within the same 900****km^2^ grid, we had 982 and 896 observations to model lynx and bobcat distribution, respectively. The occupancy model for lynx showed a wide swath of high habitat suitability across central Canada, and into Alaska, the Rocky Mountains, and the northeast of the United States (Fig. 2a). High areas of habitat suitability occur in eastern Canada as well, for example in Labrador and Québec. However, this region in particular is modeled as lower suitability than the central and western parts of the lynx range. AUC value for test data (0.863) was high, indicating the model successfully discriminated presence from background locations. The model for bobcat revealed a largely uniform mid-level of habitat suitability across much of the US and southern Canada that extends into central Mexico, with a region of lower suitability in the prairie region of the US and Canada (Fig. 2b). AUC values for test data were marginally lower for bobcat than lynx (0.778), which is consistent with that expected for a generalist species [49]. Based on jackknife estimates, maximum temperature of the warmest month was the most influential variable for the Canada lynx model when considered alone, with snow depth and ecoregion also being highly influential (Fig. S1). However, the ecoregion variable decreased the performance the most when omitted, indicating it has the most information not contained within the other predictor variables. Minimum temperature of the coldest month, snow cover, and temperature seasonality were most influential for the bobcat model, with ecoregion again decreasing the performance the most when omitted (Fig. S2).

### Niche Breadth Comparison

**Figure 3:**
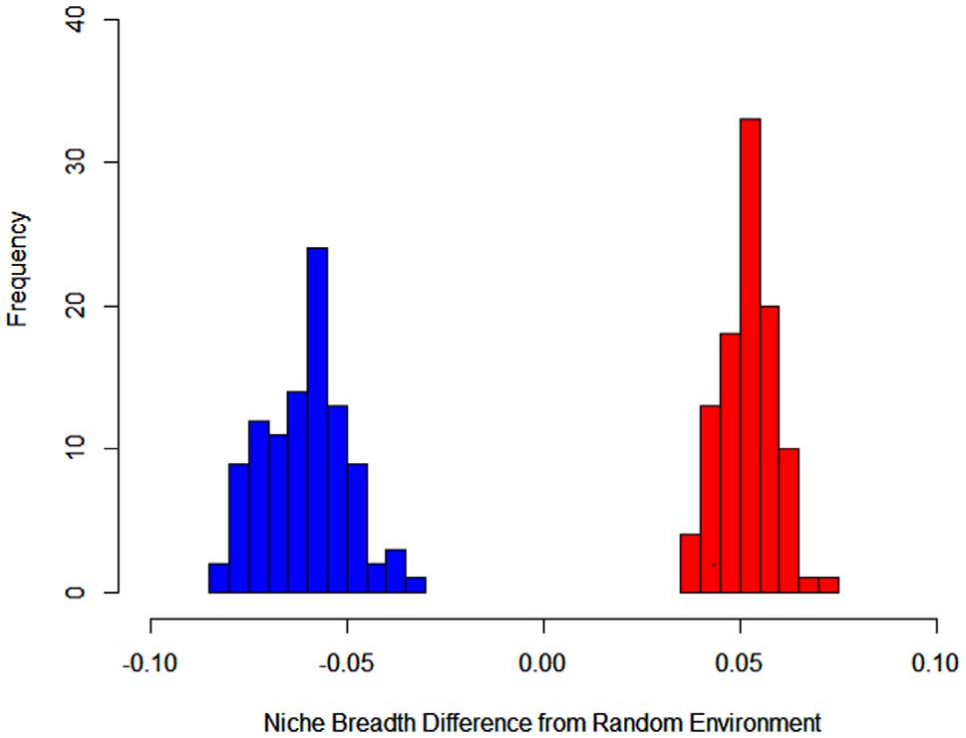
Niche breadth of Canada lynx and bobcats adjusted for background environment. The difference between the actual niche breadth of Canada lynx (blue) and bobcat (red) with the background environment. A difference of 0 would indicate the niche breadth for the species was similar to the background environment. Niche breadth was calculated based on Levin’s concentration metric.

Our niche breadth analysis revealed that lynx were the specialist species relative to bobcat. Comparisons with null models indicated that the observed niche breadth of lynx was narrower than expected if they were randomly distributed, whereas bobcats had broader niche breadth than expected compared to random (lynx: −0.061, bobcat: 0.052, *t*_198_: −88.350, *P*<0.001; Fig. 3). Bobcats therefore used a wider range of environments than lynx, after accounting for availability of different environments within the range of the species.

### Testing the Two Relationships

**Figure 4:**
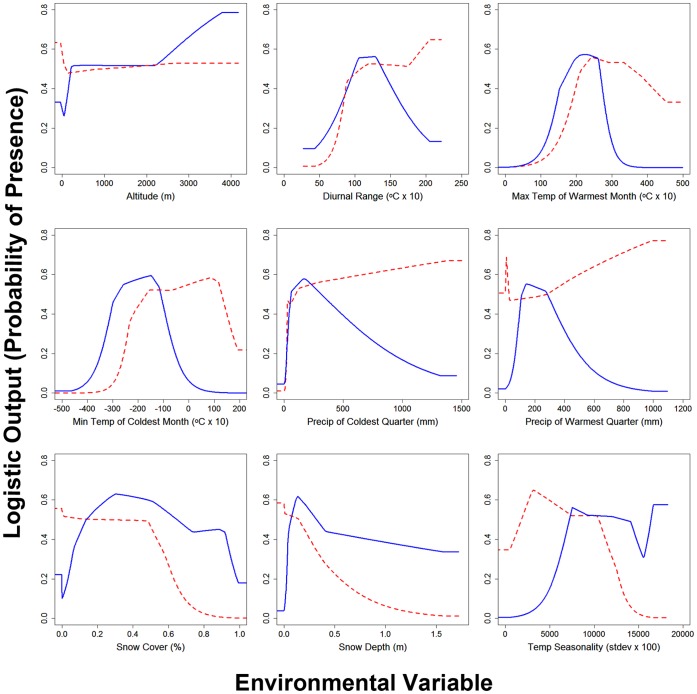
Individual response curves of the Canada lynx and bobcat for environmental variables. Response curves for Canada lynx (blue, solid line) and bobcat (red, dotted line) are given with the y axis representing probability of occurrence over the variables in units provided by the source of the data. The curves represent models developed for each species using only the corresponding variable.

**Figure 5:**
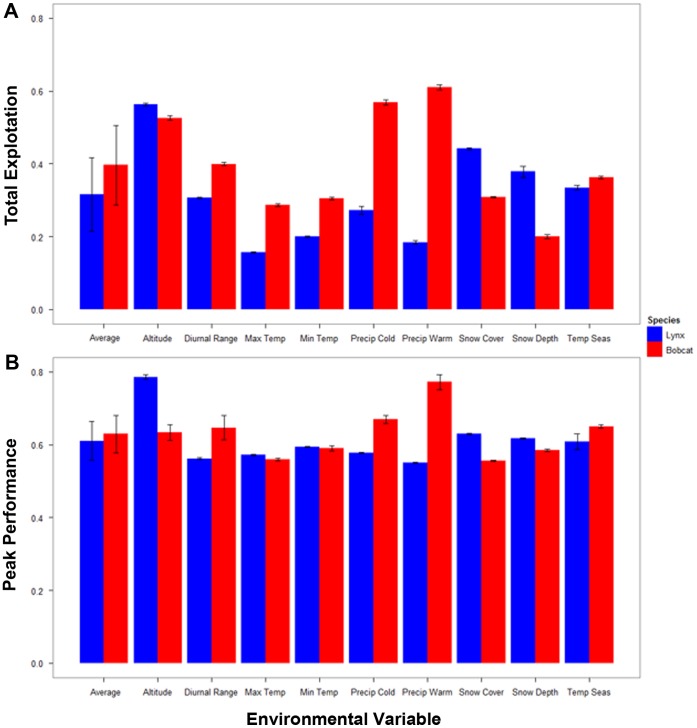
Area under the curve and peak performances of both species over each environmental variable. A) Represents the average area under the curve value for Canada lynx (blue) and bobcat (red) for the response curves for each environmental variable. B) The average peak performance value for Canada lynx (blue) and bobcat (red) for the same response curves. Confidence intervals (95% CI) that are large enough to be displayed are visible on the graph.

Values of peak performance (i.e., peak probability of occurrence) and resource utilization (i.e., area-under-the-curve) obtained from species’ response curves (Fig. 4) fails to support the hypothesized generalist-specialist dichotomy depicted in either Fig. 1a or Fig. 1b. Average values of peak performance (i.e., averaged across all 9 environmental variables) were almost identical for lynx and bobcat (average peak performance ±95% CI; lynx: 0.611±0.054, bobcat: 0.629±0.052; *t*_16_∶0.56, *P* = 0.29), which is inconsistent with Fig. 1a. In addition, total resource utilization was comparable for lynx (0.315±0.101) and bobcats (0.396±0.109; *t*_16_ = 1.26, *P* = 0.11; Fig. 5a and 5b), which is inconsistent with Fig. 1b. However, the average values of total resource utilization do trend in the direction predicted by Fig. 1b (with lower total resource utilization by lynx). Accordingly, we surmise that neither the standard model characterizing specialists and generalists nor our alternative version were particularly strong fits to the data, but our proposed alternative model (Fig. 1b) did have modest support in terms of some of the niche axes (environmental variables) under consideration.

Note that the relationship between peak performance and resource use was variable when single environmental factors were compared between species (Fig. 5a and 5b). When considering the top two predictor variables that exerted the strongest influence on the distribution of each species, lynx and bobcat each had higher resource use in one of the variables that scored high for the other species. Bobcats had higher total resource use for maximum temperature of the warmest month (lynx: 0.157±0.001, bobcat: 0.287±0.004) and minimum temperature of the coldest month (lynx: 0.200±0.001, bobcat: 0.304±0.003), whereas Canada lynx had the highest for snow cover (lynx: 0.442±0.003, bobcat: 0.308±0.002) and snow depth (lynx: 0.379±0.016, bobcat: 0.201±0.005). However, it is notable that total resource use was higher for bobcat in 4 of the remaining 5 variables, diurnal range (lynx: 0.306±0.002, bobcat: 0.399±0.004), precipitation of the coldest quarter (lynx: 0.271±0.011, bobcat: 0.569±0.007), precipitation of the warmest quarter (lynx: 0.185±0.005, bobcat: 0.610±0.007), and temperature seasonality (lynx: 0.334±0.007, bobcat: 0.363±0.003). In contrast, for the altitude variable lynx had a higher total use (lynx: 0.563±0.004, bobcat: 0.526±0.006).

For peak probability of occurrence, lynx had the higher peak in the four top performing predictor variables, although values for the two temperature-related variables were nearly identical. Lynx had higher peak performance for maximum temperature of the warmest month (lynx: 0.572±0.001, bobcat: 0.560±0.003), minimum temperature of the coldest month (lynx: 0.594±0.001, bobcat: 0.590±0.007), snow cover (lynx: 0.630±0.002, bobcat: 0.556±0.002), snow depth (lynx: 0.617±0.002, bobcat: 0.585±0.004), as well as altitude (lynx: 0.786±0.006, bobcat: 0.633±0.021). Bobcats had the higher peak in the remaining four variables, diurnal range (lynx: 0.562±0.002, bobcat: 0.647±0.032), precipitation of the coldest quarter (lynx: 0.578±0.002, bobcat: 0.670±0.011), precipitation of the warmest quarter (lynx: 0.551±0.002, bobcat: 0.772±0.021), and temperature seasonality (lynx: 0.608±0.022, bobcat: 0.651±0.005). Taken together, only two variables (max. temp. of warmest month, min. temp. of coldest month) corresponded to the proposed alternative view of niche breadth (Fig. 1b), with the specialist lynx having similar peak performance and lower total resource use compared to the generalist bobcat. In contrast, none of the patterns of resource use and performance seen in the individual variables conformed to the standard model (Fig. 1a). It is important to note that environmental variables generally differed from the two predicted relationships in that typically one species had both a higher total exploitation and peak performance for the same variable. As well, peak performance never reached 0.8 for either species meaning that neither was ever close to reaching a performance threshold.

The ecoregion variable was not used in these results because it was categorical and thus could not be evaluated on the standard niche breadth scale. However, bobcats displayed greater total resource utilization, with a >0.5 probability of presence in 9 of the 15 regions compared to only 3 of 15 for lynx. Bobcats also had the higher peak in performance in comparison with lynx (0.684, 0.631 for bobcat and lynx, respectively). This pattern also fails to conform to either of the two hypothesized models of resource use.

## Discussion

Our analysis indicates that lynx and bobcats differ substantially in their overall niche breadth but fail to conform to the traditional model of relative niche breadth differences between specialists and generalists (Fig. 1a). Of the nine environmental variables under consideration, none conformed to similar total resource exploitation but higher peak performance over a narrow range by the specialist lynx. However, lynx and bobcats do not fully match our predicted alternative model of niche breadth (Fig. 1b), with only the average values across all environmental variables (and two of the individual variables) indicating that the specialist had a trend toward lower total resource utilization than the generalist, while achieving a similar peak performance. Importantly, our results suggest that the relative difference in niche breadth between specialist and generalist species is largely dependent on the particular response variable under consideration. Because use of SDMs is an appropriate technique for quantifying niche breadth dynamics [21], including among species at higher trophic levels [25], our test is a robust assessment of the standard model of niche breadth dynamics and generalist-specialist differentiation. The inconsistency between our predicted and observed results reveals the challenges in rigorously addressing these questions in species having naturally complex life histories and interactions with the environment.

Generally, the same species had both the highest performance and the greatest total resource utilization. Bobcats peaked at a higher performance value and resource complexity associated with utilization than Canada lynx for the majority of variables; these results are similar to other studies demonstrating that some species are the jack of all trades and the masters of some [50], [51]. However, lynx displayed the larger breadth and higher performance over a select few variables, indicating the importance of the niche axis considered. These data suggest a need to re-visit assumptions regarding how specialist and generalist use resources, and may require alternative explanations for the coexistence or evolution of these two types of species. Notably, although our alternative model failed to fully explain many of the observed patterns of resource use between specialists and generalists, it performed better than the traditional model, and is deserving of additional testing.

The comparison of niche breadth between the two species using output from the species distribution models (our test to determine if we were justified in calling bobcats “generalists” and lynx “specialists”) demonstrated that bobcats were utilizing a larger breadth of environments than would be expected based on random use of the available environment, whereas Canada lynx displayed the opposite pattern. These results were consistent with observations made for these two species in more localized field studies (see Introduction), but this is the first time they have been compared directly and across their entire range. Moreover, use of null models to correct for differences in environmental variability between lynx and bobcat ranges, suggests that these patterns represent real differences in niche breadth between the species [47]. Our use of null models in determining differences in niche breadth may form the basis for a novel and objective way to define “specialists” and “generalists”; to date such terms have been applied largely in an ad hoc manner without strong justification (e.g., [52]). From our comparison with random null models, we infer that generalists have a greater breadth of resource use than would a hypothetical organism selecting habitat/resources in proportion to their availability on the landscape (e.g., by selecting both common and rare environments), and are therefore capable of exploiting a wide-range of environments. In contrast, specialists have a narrower breadth of habitat/resource use than would an organism distributed randomly on the landscape. Moreover, this approach would enable species to be classified along a continuum, without the need to make categorical definitions of specialist or generalist, by simply analyzing where species are located in relation to the null expectation. We therefore recommend that future studies at large scales seeking to distinguish between species with broad and narrow niches should adopt a more objective rule for binning (e.g., [10]).

Other studies that have supported the “master of some” hypothesis showed that generalists have higher maximum resource use compared to specialists, even for variables that are supposed to be the primary resources for the specialist (e.g., [15], [50]). For example, the specialist antlion larvae, *C. lineosa*, had a lower performance level in the sand type it specializes in than the generalist species, *M. hyalinus* [15]. These results contrast sharply with ours because the higher resource use and peak performance of bobcats for variables not related to snow is consistent with the ecological aspects of specialization in lynx, which have morphological features allowing for improved survival in deep and soft snow [38], [53]. Therefore, our results suggest that specialist species do not necessarily tolerate a narrower breadth for each resource gradient relative to generalists, but rather have a wider breadth and higher performance value within a smaller number of resources or environmental axes. Such species may be able to coexist with generalist competitors because they can outperform generalists on those few resource axes, and therefore occupancy and abundance will be strongly linked to areas where those variables occur. Since specialist presence is strongly linked to select variables, the density of these species typically would be lower on the landscape as there are fewer habitats that they perform highly in, and therefore could only support a smaller population. A generalist species, whose performance is lower within these select few environments, will therefore, be less likely to occur in areas where these resources or environmental gradients predominate, but should be more abundant in a wider variety of locations. This observation is supported for our study species, as Canada lynx tend to have lower population densities than bobcats [54].

The defining feature of a generalist may not always be driven by a trade-off between a wide tolerance to resource gradients and maximum performance within that gradient, but rather that a greater variety of resource or environmental axes can be tolerated and utilized at a high level of performance. This suggests that to fully understand distinctions between specialists and generalists, a wide spectrum of environmental axes should be examined simultaneously. If generalists follow the pattern of a wider tolerance of environmental variables, that is not hindered by a lower peak performance, such species would be better able to invade new geographic areas and expand range limits [50]. Similarly, generalist species should be favoured during times of environmental change while the more specialised species are favoured during periods of homogeneity [5], [6]. For example, areas where snow cover and depth is consistent year to year would be most suitable for Canada lynx. Generalists should be less vulnerable to extinction on a geological time scale than specialists [55], given that they perform well on a greater variety of resource axes.

Studies comparing the responses of specialists and generalists to resource gradients have varied greatly in their results depending on species and methods used [15], [56], [57]. Our study provides a potential explanation for the apparent differences between studies, as our results clearly show that the tradeoff between resource exploitation and performance depends greatly on the particular resource axis under consideration. This reinforces the notion that a better understanding of specialist and generalist ecology will necessitate a focus on multiple resource axes. Consistency in approaches is necessary to effectively compare between species across taxa and study systems.

Our work focused on “indirect” environmental gradients, which are removed from the actual resource axes. For example, snow cover and snow depth indirectly represented access to snowshoe hare, as well as serving as a surrogate for overall physiological limits of terrestrial carnivores. The findings of this study would be strengthened by additional work examining response of lower trophic level species to direct environmental gradients, to document whether observed patterns in predators reflect those of prey species. Regardless, our results indicate that patterns of resource exploitation by specialist and generalist predators can be inferred from environmental variables even without specific information on their prey, but we do acknowledge more generally that factors differentiating specialists from generalists are not fully understood and are more complex than previously thought. Given the popularity of separating species into those with narrow or broad niche breadth to answer questions in community ecology and conservation biology [9]–[11], it is important to rigorously re-evaluate niche dynamics, and ultimately, the ecological role of generalists and specialist species.

## Supporting Information

Figure S1 **Jackknife of regularized training gain for** ***Lynx canadensis*** **.** Jackknife of regularized training gain for Canada lynx which indicates the influence of each variable in the model as well as the amount the model performance is reduced when the variable is omitted. Values shown are averages over replicated runs.
(TIF)

Figure S2 **Jackknife of regularized training gain for** ***Lynx rufus*** **.** The Jackknife of regularized training gain for bobcat which indicates the influence of each variable in the model as well as the amount the model performance is reduced when the variable is omitted. Values shown are averages over replicated runs.
(TIF)

Figure S3 **Jackknife of regularized training gain for** ***Lynx canadensis*** **of the 10 km grid model.** The Jackknife of regularized training gain for Canada lynx which indicates the influence of each variable in the model as well as the amount the model performance is reduced when the variable is omitted. Values shown are averages over replicated runs.
(TIF)

Figure S4 **Jackknife of regularized training gain for** ***Lynx canadensis*** **of the 20 km grid model.** The Jackknife of regularized training gain for Canada lynx which indicates the influence of each variable in the model as well as the amount the model performance is reduced when the variable is omitted. Values shown are averages over replicated runs.
(TIF)

Figure S5 **Jackknife of regularized training gain for** ***Lynx rufus*** **of the 10 km grid model.** The Jackknife of regularized training gain for bobcat which indicates the influence of each variable in the model as well as the amount the model performance is reduced when the variable is omitted. Values shown are averages over replicated runs.
(TIF)

Figure S6 **Jackknife of regularized training gain for** ***Lynx rufus*** **of the 20 km grid model.** The Jackknife of regularized training gain for bobcat which indicates the influence of each variable in the model as well as the amount the model performance is reduced when the variable is omitted. Values shown are averages over replicated runs.
(TIF)

Table S1 **Source of museum specimen records and recent state/province harvest records.**
(PDF)
