# Supplementary figures and images for: Reconsidering the Specialist-Generalist Paradigm in Niche Breadth Dynamics: Resource Gradient Selection by Canada Lynx and Bobcat
Source: PLoS One. 2012 Dec 7;7(12):e51488. doi: 10.1371/journal.pone.0051488 (PMC3517500; doi:10.1371/journal.pone.0051488)

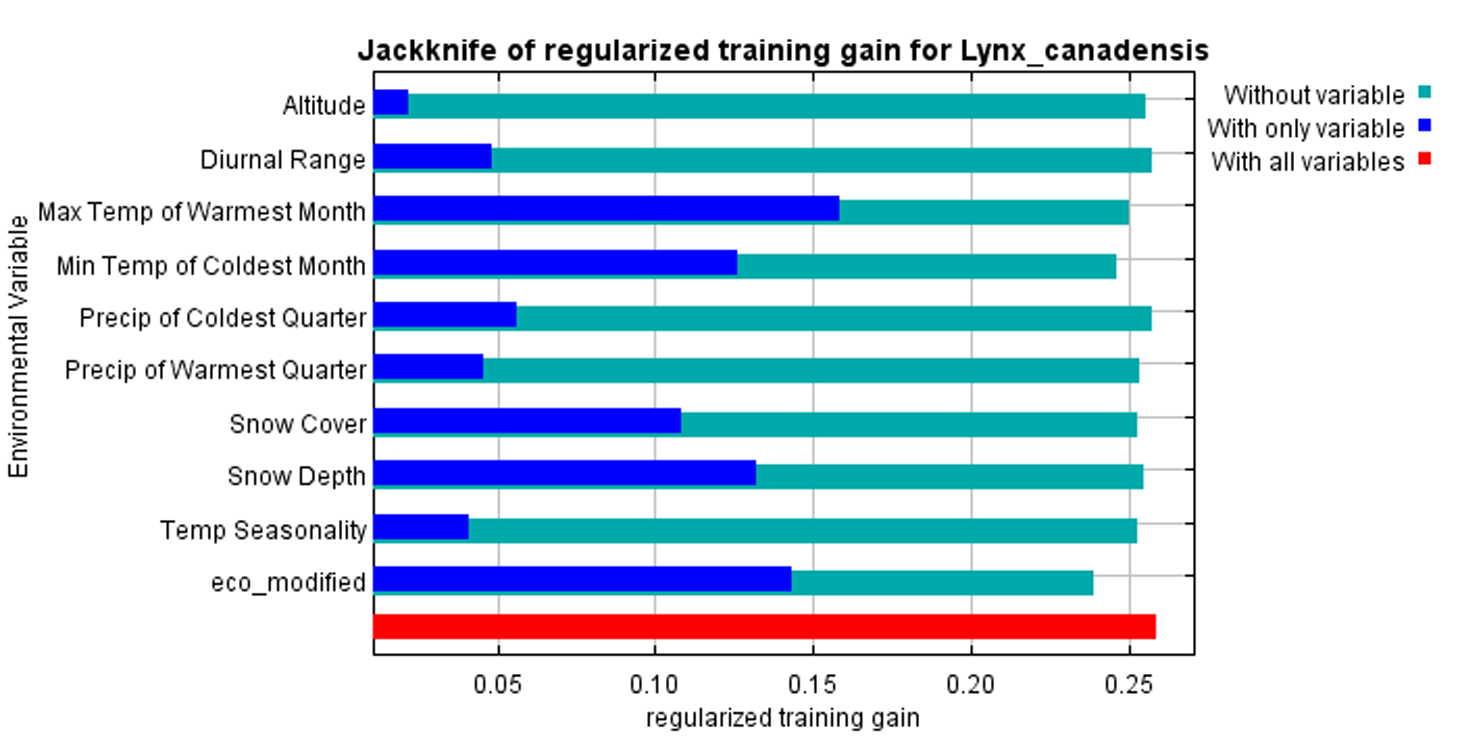

Supplement: Figure S1 — Jackknife of regularized training gain for Lynx canadensis . Jackknife of regularized training gain for Canada lynx which indicates the influence of each variable in the model as well as the amount the model performance is reduced when the variable is omitted. Values shown are averages over replicated runs. (TIF) [file pone.0051488.s001.tif]

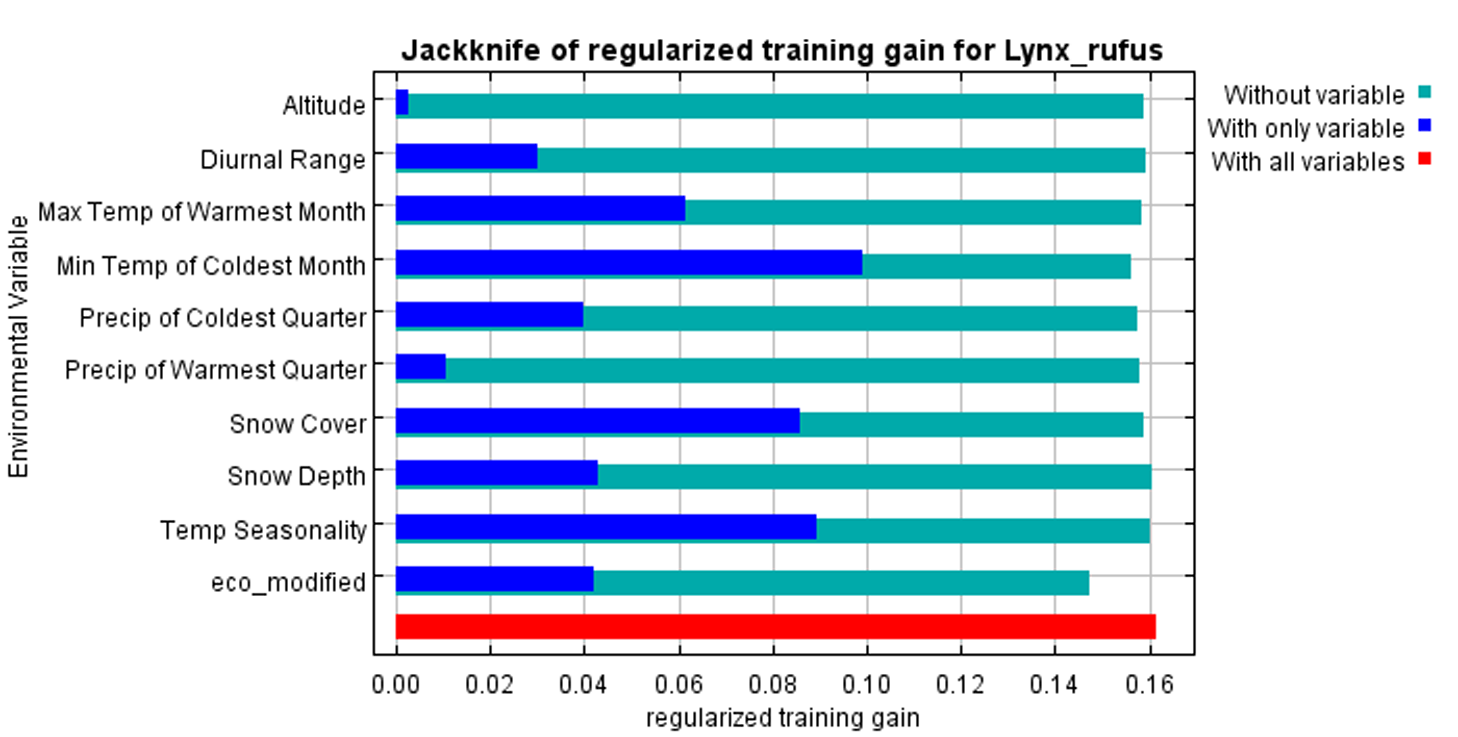

Supplement: Figure S2 — Jackknife of regularized training gain for Lynx rufus . The Jackknife of regularized training gain for bobcat which indicates the influence of each variable in the model as well as the amount the model performance is reduced when the variable is omitted. Values shown are averages over replicated runs. (TIF) [file pone.0051488.s002.tif]

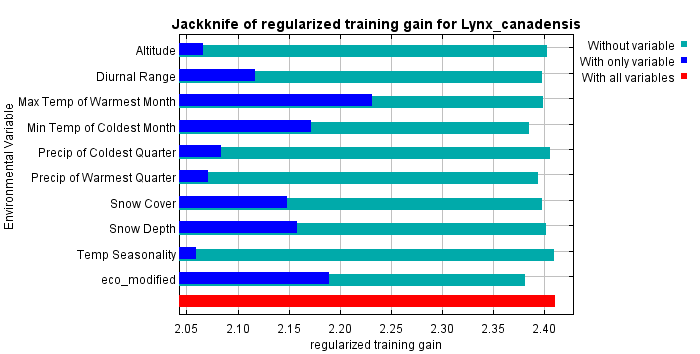

Supplement: Figure S3 — Jackknife of regularized training gain for Lynx canadensis of the 10 km grid model. The Jackknife of regularized training gain for Canada lynx which indicates the influence of each variable in the model as well as the amount the model performance is reduced when the variable is omitted. Values shown are averages over replicated runs. (TIF) [file pone.0051488.s003.tif]

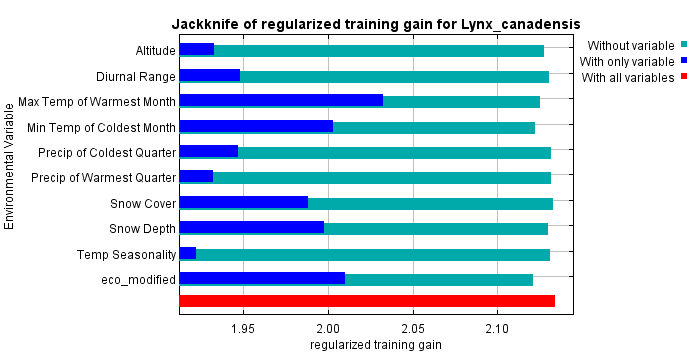

Supplement: Figure S4 — Jackknife of regularized training gain for Lynx canadensis of the 20 km grid model. The Jackknife of regularized training gain for Canada lynx which indicates the influence of each variable in the model as well as the amount the model performance is reduced when the variable is omitted. Values shown are averages over replicated runs. (TIF) [file pone.0051488.s004.tif]

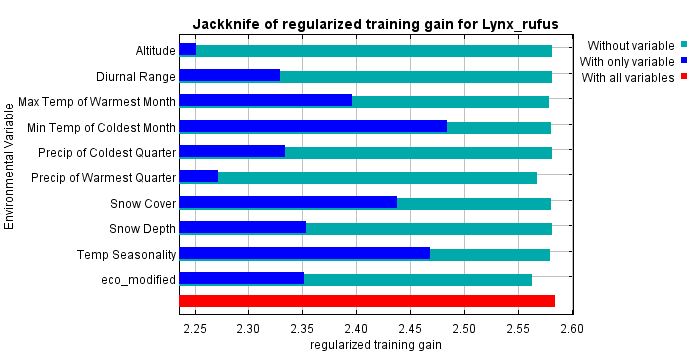

Supplement: Figure S5 — Jackknife of regularized training gain for Lynx rufus of the 10 km grid model. The Jackknife of regularized training gain for bobcat which indicates the influence of each variable in the model as well as the amount the model performance is reduced when the variable is omitted. Values shown are averages over replicated runs. (TIF) [file pone.0051488.s005.tif]

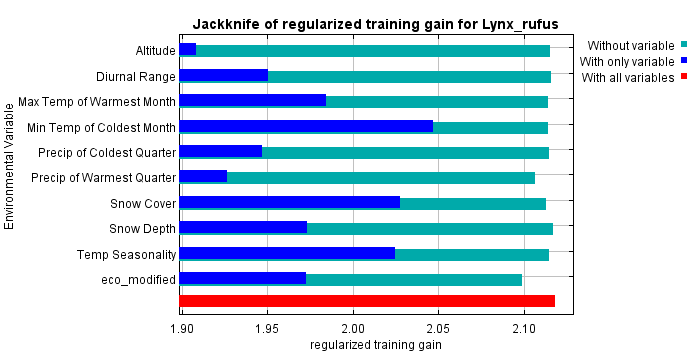

Supplement: Figure S6 — Jackknife of regularized training gain for Lynx rufus of the 20 km grid model. The Jackknife of regularized training gain for bobcat which indicates the influence of each variable in the model as well as the amount the model performance is reduced when the variable is omitted. Values shown are averages over replicated runs. (TIF) [file pone.0051488.s006.tif]
