# Supplementary material for: Reconsidering the Specialist-Generalist Paradigm in Niche Breadth Dynamics: Resource Gradient Selection by Canada Lynx and Bobcat
Source: PLoS One. 2012 Dec 7;7(12):e51488. doi: 10.1371/journal.pone.0051488 (PMC3517500; doi:10.1371/journal.pone.0051488)
Supplement: Table S1 — Source of museum specimen records and recent state/province harvest records. (PDF) [file pone.0051488.s007.pdf]

Table S1: Source of museum specimen records and recent state/province harvest records, all data were accessed or received in 2011.

Type	Location
**Museum**	Museums listed in Mammal Networked Information System (MaNIS) (www.manisnet.org)
	Museums listed in CONABIO (www.conabio.gob.mx)
	Philip L. Wright Zoological Museum, Missoula, Montana
	Virginia Museum of Natural History, Martinsville, Virginia
	North Carolina Museum of Natural Sciences, Raleigh, North Carolina
	Carnegie Museum of Natural History, Pittsburgh, Pennsylvania
	Bell Museum of Natural History, Minneapolis, Minnesota
	Museum of Cultural and Natural History,
	Cleveland Museum of Natural History, Cleveland, Ohio
	University of Arkansas Collections, Fayettevelle, Arkansas
	University of Alabama Museums, Tuscaloosa, Alabama
	University of Northern Iowa Museums, Cedar Falls, Iowa
	Royal British Columbia Museum, Victoria, British Columbia
	Manitoba Museum, Winnipeg, Manitoba
	Royal Saskatchewan Museum, Regina, Saskatchewan
	Royal Alberta Museum, Edmonton, Alberta
	Idaho Museum of Natural History, Pocatello, Idaho
	Connecticut State Museum of Natural History, Storrs, Connecticut
**Harvest**	Ontario
	Saskatchewan
	Quebec
	British Columbia
	Alaska
